# Role reconstruction among double-qualified nursing educators in the generative AI era: a qualitative study

**DOI:** 10.3389/fmed.2026.1776845

**Published:** 2026-04-10

**Authors:** Mingyan Shen, Shuqi Xue, Fangchi Liu, Jie Lang

**Affiliations:** 1 Shulan International Medical College, Zhejiang Shuren University, Hangzhou, China; 2 Institute of AI-Empowered Full Lifecycle Care, Zhejiang Shuren University, Hangzhou, China; 3 Hangzhou Normal University, Hangzhou, China

**Keywords:** clinical reasoning, double-qualified nursing educators, generative AI, nursing education, qualitative research, social constructivism

## Abstract

**Background:**

Generative AI (GenAI) is rapidly integrating into nursing education, acting as a novel tool for knowledge mediation. While it offers new learning opportunities, its application risks disrupting essential social interactions and clinical contextualization. Double-qualified nursing educators (DQNEs) play a pivotal role in navigating this technological shift.

**Objectives:**

This study adopts a social constructivist framework to examine the pedagogical functional boundaries of GenAI in nursing education and to analyze how DQNEs reconstruct their roles to facilitate knowledge co-construction and clinical meaning-making.

**Design:**

A qualitative study using semi-structured focus group interviews.

**Methods:**

The study was conducted at a medical college in eastern China. Eighteen DQNEs with experience in GenAI integration participated. Data were collected through three focus group discussions and analyzed using thematic analysis to identify key themes.

**Results:**

Three key themes emerged: (1) Application value of GenAI in nursing education, where GenAI served as a scaffolding tool to trigger cognitive conflict, support differentiated instruction, and bridge theory with simulated scenarios; (2) Core obstacles to GenAI application, revealing challenges of tool dependency displacing human interaction, erosion of teacher authority, and institutional ambiguity; and (3) Adaptive pedagogical strategies for anchoring learning in clinical practice, where teachers employed deconstruction and reconstruction strategies and enforced social rules to anchor AI-generated plans in clinical practice.

**Conclusion:**

GenAI functions as a double-edged mediating tool that can expand the zone of proximal development but also threatens to social learning. To mitigate epistemic risks, DQNEs must evolve from information transmitters to contextual anchors, guiding students to validate GenAI outputs against clinical reality.

## 1 Introduction

The rapid evolution of generative artificial intelligence (GenAI) is fundamentally reshaping how knowledge is produced and disseminated in higher education. As GenAI demonstrates increasing proficiency in natural language understanding, scenario generation, and logical reasoning, it has evolved from a passive support tool into an active agent capable of participating in knowledge co-construction and meaning-making processes (1–3). Current research indicates that this technological transformation is driving professional education away from traditional unidirectional knowledge transmission toward educational ecosystems that emphasize human-AI collaboration and dynamic generation (4). Within this emerging ecosystem, nursing education confronts distinctive opportunities and challenges. The discipline inherently requires learners to master interdisciplinary theoretical frameworks while developing practical wisdom for precise judgment in rapidly evolving clinical contexts. Recent studies indicate that GenAI can construct highly realistic virtual clinical environments through its powerful generative and simulation capabilities. This has been shown to effectively address clinical teaching resource shortages and provide new possibilities for transcending traditional spatiotemporal constraints (5, 6).

Despite significant technological potential, academic concerns regarding application risks have intensified (7). Although GenAI generated virtual scenarios can simulate clinical appearances, they often struggle to accurately capture the nuanced humanistic ethics and complex decision-making logic inherent in healthcare practice. Empirical studies have identified “machine hallucinations” or decontextualized recommendations in AI-generated content (8, 9). This subtle discrepancy between “highly realistic simulation” and “authentic clinical practice” can easily become a cognitive trap that misleads novice learners without professional guidance grounded in clinical acuity. Faculty often cannot identify clinical detail errors in GenAI outputs, while purely clinical experts may lack the pedagogical design capabilities to transform these into teaching materials. Consequently, in contexts of deep technological intervention, Double-qualified nursing educators (DQNEs) have become increasingly important. In this study, DQNEs refer to professionals who possess both a higher education teaching qualification and a nursing practice license (10, 11). Unlike previous research perspectives that focused primarily on their clinical skills, this study posits that DQNEs function not merely as implementers of digital teaching but as mediators of clinical judgment and facilitators of meaning-making in human–machine collaboration. Through this mediating role, DQNEs help maintain the alignment of technological applications with core nursing professional values.

However, a research gap remains regarding how to reconstruct these professional roles and elucidate the complex interaction mechanisms between GenAI, DQNEs, and nursing undergraduates (12). Social constructivism theory provides a robust lens for understanding this complex process. The theory posits that knowledge is dynamically generated through continuous negotiation and reflection during social interaction within specific cultural contexts (13, 14). In AI-mediated classrooms, technology alters existing interaction structures, transforming learning into a triadic interaction among teachers, students, and GenAI. Although research on GenAI applications in education is expanding rapidly, existing literature predominantly focuses on technology acceptance, tool performance evaluation, or student satisfaction surveys (15–17). Systematic empirical examination remains limited regarding how GenAI transforms the social construction of nursing knowledge and how DQNEs reshape their professional roles to mitigate cognitive risks in new technological environments.

### 1.1 Aim

Building on this background, this study adopts social constructivism as its theoretical framework to examine the pedagogical functional boundaries of GenAI in nursing education and analyze the role reconstruction pathways for DQNEs under technological intervention. This study addresses three core research questions:

1. How does GenAI serve as a mediating tool to support knowledge co-construction and clinical meaning-making in nursing education?
2. In what ways does GenAI integration disrupt or reshape the essential interactional structures, specifically teacher-student, student-student, and learner-practice interactions?
3. How can DQNEs redefine their professional roles, and what institutional supports are required to address these interactional challenges?

## 2 Materials and methods

### 2.1 Study design

This study employed a qualitative exploratory design. It used focus group interviews to capture the breadth and depth of nursing educators’ experiences. The Consolidated Criteria for Reporting Qualitative Research (COREQ) checklist was followed (18).

### 2.2 Settings

The study was conducted at Shulan International Medical College, Zhejiang Shuren University. The college is affiliated with a teaching hospital that employs over 70 DQNEs. These educators are responsible for a diverse curriculum, including professional ethics, internal medicine nursing, surgical nursing, pediatric nursing, critical care nursing, psychiatric nursing, rehabilitation nursing, traditional Chinese medicine nursing, and nursing management. Collectively, they deliver more than 2,000 teaching hours annually.

### 2.3 Participants and sampling

In October 2025, a purposive sampling strategy was employed to recruit participants from a university in Zhejiang Province. Based on the principle of maximum variation, 18 DQNEs were selected to ensure diverse perspectives regarding the integration of GenAI in nursing education.

The demographic profiles of the participants are presented in Table 1. All participants were female, ranging in age from 29 to 47 years. Their teaching experience varied from 3 to 12 years, ensuring a mix of novice and experienced educators. To capture a holistic view of the curriculum, the participants were selected from a wide range of teaching specialties, including foundational nursing courses, medical surgical nursing, maternal and child health nursing, mental health nursing, emergency and critical care nursing, as well as applied and professionally oriented courses such as nursing informatics, ethics, and advanced nursing assessment. The 18 participants were divided into three focus groups, with six participants in each group. This group size is consistent with recommendations for focus group research, and data saturation was reached when no new themes emerged in the third focus group (19). The grouping strategy aimed to mix teachers from different specialties to stimulate interdisciplinary dialogue regarding GenAI application (20).

**TABLE 1:** Demographic characteristics of the participants.

Characteristic	*n* (%) or mean ± SD
Age (years)	38.1 ± 6.4
Teaching experience (years)	5.2 ± 2.5
Gender	
Female	18 (100.0)
Male	0 (0.0)
Clinical role	
Head nurse	10 (55.6)
Senior nurse	8 (44.4)
Professional title	
Intermediate	8 (44.4)
Senior	10 (55.6)
Teaching specialty	
Foundational nursing courses	3 (16.7)
Medical–surgical nursing	5 (27.8)
Maternal and child health nursing	3 (16.7)
Mental health nursing	1 (5.5)
Emergency and critical care nursing	1 (5.5)
Applied and professionally oriented courses	5 (27.8)

Inclusion criteria: (1) currently employed as a DQNE at the university; (2) actively teaching at least one undergraduate nursing core course during the study period; (3) had prior experience or active attempts in incorporating GenAI tools into their teaching design, case preparation, or assessment; (4) willingness to participate in the focus group discussions and provide informed consent.

The GenAI experience was assessed during recruitment through self-report. Participants were asked to provide one example of GenAI use in teaching related activities. Eligibility was confirmed by the research team based on these responses. Educators were excluded if they were unable to participate in the focus group interviews or declined to provide informed consent.

### 2.4 Data collection

Data were collected from October to November 2025 through semi-structured focus group interviews. This method facilitated dynamic interaction and allowed for a deeper exploration of collective experiences regarding GenAI integration in nursing education. The interview guide was grounded in Social Constructivism Theory and focused on scaffolding, social interaction, and situated learning to examine how GenAI mediates knowledge construction and clinical reasoning.

The interviews explored the pedagogical boundaries of GenAI and the evolving role of educators. Questions addressed the current landscape of GenAI usage in nursing education, how teachers utilized GenAI as a scaffolding tool in the pedagogical process, and the interactions between GenAI and teaching. Participants also discussed the alignment between GenAI knowledge and clinical reality. The complete interview guide is provided in the Supplementary File 1.

### 2.5 Data analysis

Audio recordings were transcribed within 24 h by two researchers (XSQ and LFC) using the offline transcription tool iFLYTEK SR302 smart recorder. Transcripts were cross-verified with field notes to ensure accuracy and completeness.

We employed thematic analysis following Braun and Clarke’s framework, and coding was performed using QualCoder software (21, 22). The research team began by reading and rereading the transcripts, then coded them line by line to identify recurring features related to GenAI integration in nursing education. Related codes were grouped into potential themes. Through team discussion and further data review, we ensured that each theme was well-supported by the data and accurately reflected the core aspects of GenAI usage in nursing pedagogy. We clearly defined and named the themes, refining them to ensure each theme reflected the data and research questions. In the final report, we presented the definitions and data extracts for each theme, providing a clear narrative that linked the findings to the research questions. We also incorporated member checking by sharing preliminary findings with two participants to confirm the accuracy and relevance of our interpretations. Their feedback was used to refine the final report.

### 2.6 Reflexivity

The research team consisted of scholars with diverse academic backgrounds relevant to nursing education and qualitative research. All authors had received formal training in qualitative research methodologies and possessed experience in conducting independent qualitative studies.

Each focus group had a moderator and an observer to facilitate discussion and monitor group dynamics. The interviews were conducted in private rooms without interruption. Each interview lasted around 60–90 min, with flexibility to extend them as needed. The researchers maintained neutrality, clarifying any unclear responses by asking follow-up questions. To enhance the credibility of the findings, the team engaged in reflexivity, continuously reflecting on their roles, assumptions, and potential biases during data analysis and interpretation.

## 3 Results

**FIGURE 1:**
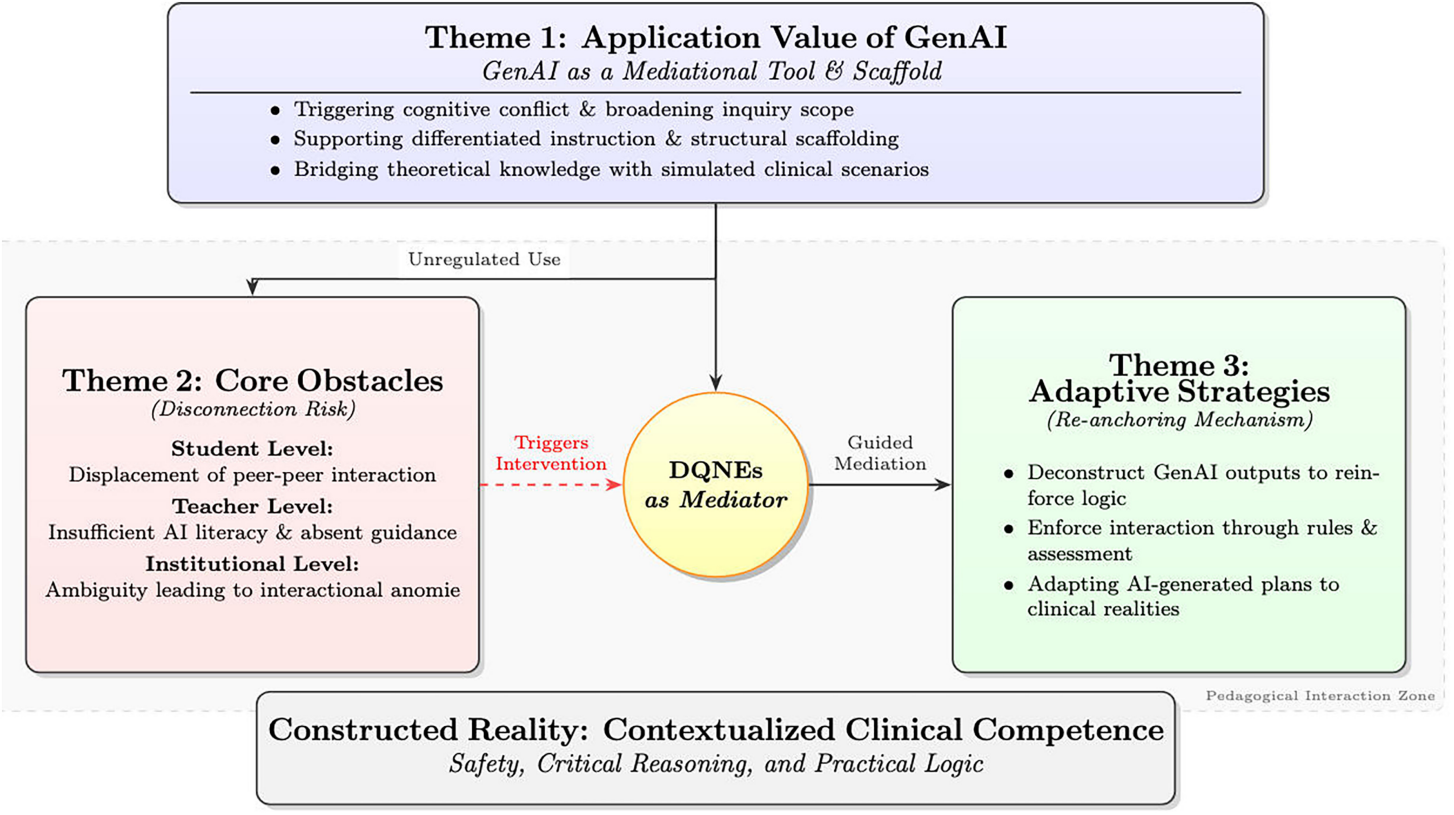
The generative AI (GenAI) integrated nursing clinical knowledge construction framework.

Three main themes were identified from participant narratives guided by a social constructivist framework: (1) application value of GenAI in nursing education; (2) core obstacles to GenAI application; and (3) adaptive pedagogical strategies for anchoring learning in clinical practice. In addition, a conceptual framework is constructed to organize these themes (Figure 1).

### 3.1 Theme 1: application value of GenAI in nursing education

Participants described GenAI not only as an information repository, but as a novel mediational tool that altered the starting point and breadth of knowledge construction. By generating diverse and sometimes conflicting information, GenAI effectively triggered student inquiry and shifted the instructional focus from information retention to higher-order clinical reasoning.

#### 3.1.1 Triggering cognitive conflict and broadening inquiry scope

From a social constructivist perspective, problems function as the starting point for knowledge construction. Interview data indicated that GenAI frequently provided clinical cases and structured knowledge points that contained internal inconsistencies, which triggered cognitive conflict and prompted dialogue between teachers and students.

One teacher described a teaching session on post-thrombolysis heart rate monitoring in which GenAI generated three different monitoring intervals. “*Faced with these discrepancies, students immediately questioned the validity of the responses and organized themselves to consult clinical guidelines”* (*P9 Internal Medicine Nursing*). The teacher noted that this process produced deeper understanding than direct explanation.

In addition, GenAI efficiently supported lower-order cognitive tasks, including information retrieval and conceptual organization, thereby reducing instructional time devoted to basic content delivery. This shift allowed teachers to allocate more time to higher-order learning activities. Another teacher reported that “*when GenAI rapidly summarized the Wagner classification for diabetic foot care, classroom time was redirected toward analyzing how nursing plans should be adapted according to individual vascular conditions*” (*P10 Nursing Assessment*).

Overall, GenAI functioned as a supportive learning tool that reduced routine cognitive demands and facilitated more intensive interaction focused on clinical reasoning and contextualized application.

#### 3.1.2 Supporting differentiated instruction and structural scaffolding

Data showed that GenAI supports stratified teaching by generating adaptive resources. For students with weaker foundational knowledge, GenAI played a crucial role in lowering the cognitive threshold by building initial knowledge frameworks. One instructor noted that by utilizing GenAI as a visual and structural aid, students could establish a baseline understanding before the teacher introduced clinical nuances.

*“when students confused Babinski and Oppenheim signs, allowing them to search via GenAI allows them to create a clear knowledge framework. On this basis, I supplement the differentiation points, accelerating their understanding.” (P8 Neurology Nursing)*

Conversely, for advanced learners GenAI was utilized to generate multi-disciplinary cases that challenged their clinical reasoning. Teacher 6 described using GenAI to create a scenario involving pediatric urinary tract infection complicated by Type 1 diabetes,

*“The case covered three difficulties, requiring students to integrate pediatric nursing, endocrinology, and pharmacology. I then supplemented with practical clinical insights, such as how to dynamically adjust blood glucose monitoring frequency and which renal function indicators to watch after specific antibiotic use. This helped them further understand the knowledge application.” (P6 Pediatrics Nursing)*

GenAI provides structure then teacher fills context mode effectively reduced the cognitive load for beginners while providing necessary complexity for advanced learners, thereby promoting meaningful knowledge construction across different competency levels.

#### 3.1.3 Bridging theoretical knowledge with simulated clinical scenarios

Double-qualified nursing educators used GenAI to embed clinical scenarios into classroom teaching, guiding students to compare AI-generated recommendations with practical application and reflective discussion.

*“Students had GenAI generate a “Day 1 post-cholecystectomy” case. GenAI suggested encouraging ambulation within 6 h, but failed to consider individual differences. I supplemented this by explaining that clinical practice requires assessing consciousness, blood pressure stability, and orthostatic hypotension risks. I also displayed real nursing records to show critical details GenAI cannot provide, such as dynamic vital sign changes, helping students understand the clinical logic behind nursing decisions.” (P13 Surgery Nursing)*

Similarly, another teacher utilized this model to highlight safety risks in neurosurgical rehabilitation.

*“I introduced a real case: on the third day after surgery, a patient experienced syncope and fell during rehabilitation training because orthostatic hypotension was not monitored, resulting in a fracture. This case helped students translate abstract rehabilitation theory into safe and actionable clinical behavior” (P17 Surgery Nursing)*

Furthermore, seven teachers consistently noted that GenAI often lacked clinical detail, requiring teacher intervention for correction.

*“Regarding muscle strength grading, GenAI only broadly described grades 0–5, but in clinical practice, we must distinguish between proximal and distal muscle strength. For example, a stroke patient might have Grade 3 proximal strength but only Grade 1 distal strength, a difference that directly affects the rehabilitation plan.” (P8 Neurology Nursing)*

These findings indicate that while GenAI provides the necessary foundational context, the “Clinical Faculty Calibration” is essential for closing the loop on clinical thinking.

### 3.2 Theme 2: core obstacles to GenAI application

Despite the identified value, participants revealed multidimensional barriers rooted in the disruption of meaningful interaction. The convenience of GenAI frequently weakened the social learning process, resulting in knowledge construction that was detached from clinical reality.

#### 3.2.1 Student level: displacement of peer-to-peer interaction

Interviews revealed that reliance on GenAI systematically weakened individual practice and peer-peer interactions. Six teachers reported students substituting authentic fieldwork with AI generation. One teacher described an assignment requiring students to interview a postpartum woman to analyze psychological needs:

*“More than 90% of the students submitted highly identical cases. It was obvious that no field interviews were conducted. They replaced the personal experience of listening to a patient with an AI-generated summary.” (P5 Obstetrics and Gynecology Nursing)*

Second, peer collaboration mechanisms became superficial. In group tasks, the collaborative process of negotiation and debate was often replaced by individual students generating content through GenAI.

*“It is often just the group leader using GenAI to generate all the content. Other members neither participate in the literature search nor discuss operational details. They don’t even notice contradictory data within the plan.” (P1 Nursing Informatics)*

This reliance not only nullified the benefits of collaborative learning but also led to a dangerous distortion of clinical meaning. Seven teachers emphasized that this blind trust eroded students’ critical judgment regarding clinical complexity.

*“In a psychiatric nursing case study, students directly adopted the GenAI’s suggestion for “monotherapy for sleep disorders,” completely ignoring the clinical reality of polypharmacy, drug interactions, and the synergistic risks of side effects. They treated the GenAI answer as the final clinical decision.” (P7 Psychiatry Nursing)*

#### 3.2.2 Teacher level: insufficient AI literacy led to absent guidance roles

Participants acknowledged that a lack of AI literacy hindered their ability to act as effective guides. Firstly, teachers struggled with the discernment and validation of AI-generated content. This undermined their cognitive authority and stalled the collaborative learning process.

*“When students brought an AI-generated nursing plan, I could identify clinical errors in it, but I struggled to understand how to guide students to prompt it better to get the correct answer. I felt I lacked the technical knowledge to teach them effective GenAI usage.”(P10 Nursing Assessment)*

Secondly, teachers lacked the capacity to design deep, integrated learning activities.

*“I can only detect GenAI traces when grading. I haven’t designed processes that guide students to use GenAI and refine with clinical cases. GenAI remains disconnected from clinical thinking development.” (P11: Nursing Etiquette)*

Finally, this lack of design created a crisis in assessment and feedback. Teachers found it nearly impossible to distinguish between a student’s original thought and sophisticated GenAI output, which paralyzed the evaluation process.

*“Some student assignments are highly consistent with the textbook, while others are logically clear and rich with cases because they used GenAI. I don’t know how to grade them fairly.” (P5 Obstetrics and Gynecology Nursing)*

#### 3.2.3 Institutional level: ambiguity leading to interactional anomie

Effective knowledge co-construction requires clear and orderly rules. Interviews revealed that schools currently lack systematic institutional arrangements for GenAI teaching applications. First, the absence of academic norms left the boundaries of GenAI use undefined.

*“The college has no formal document defining which teaching activities can use GenAI or what constitutes academic misconduct. We can only give verbal warnings.” (P3 Thesis Advisor)*

Second, the lack of domain specific resources led to information lag. Eight teachers pointed out that students, forced to rely on general GenAI due to a lack of specialized nursing GenAI.

*“Students use general GenAI to query “rescue procedures for adult traumatic cardiac arrest,” and the results are often based on guidelines from before 2022. This information lag not only misleads learning but may also pose safety risks in future clinical practice.” (P16 Critical Care Nursing)*

Third, a lack of systematic training left teachers relying on self-exploration.

*“I don’t even know how to use GenAI to assist teaching in a standardized way. The school hasn’t organized a systematic training, so we fumble around on our own. The results are unstable, and we cannot effectively guide students toward quality knowledge co-construction.” (P12 Introduction to Nursing)*

### 3.3 Theme 3: adaptive pedagogical strategies for anchoring learning in clinical practice

Double-qualified nursing educators employed adaptive pedagogical strategies to restore the link between GenAI and clinical reality. By repositioning themselves as critical mediators, they actively worked to anchor AI-generated knowledge in the safety and complexity of patient care.

#### 3.3.1 Deconstructing GenAI outputs to reinforce clinical logic

To prevent students from accepting GenAI outputs as standard answers, teachers actively guided them to deconstruct GenAI content and reconstruct personalized understanding within a realistic clinical context. Teacher 7 implemented this during Problem-Based Learning (PBL) sessions.

*“I have students use GenAI to find nursing care plans related to adverse drug reactions in schizophrenia treatment. Then I asked if the recommended Risperidone dosage was safe for elderly patients with impaired liver and kidney function. Students compared guidelines with real cases. I shared my experience with an elderly patient who had adverse reactions from high dosage. This helped students apply GenAI information to individualized clinical decisions.” (P7 Psychiatry Nursing)*

Similarly, Teacher 9 emphasized understanding physiological mechanisms behind care decisions rather than memorizing procedures.

*“In teaching COPD care, I present the GenAI suggestion of low-flow oxygen (1–2 L/min), then guide students through the pathophysiological logic. We combine this with the clinical target of SpO2 88%–92% to clarify why we cannot blindly pursue high oxygen saturation.”(P9 Internal Medicine Nursing)*

#### 3.3.2 Enforcing social interaction through rules and assessment

To curb overreliance on GenAI and the erosion of collaboration, some DQNEs constructed interaction management mechanisms covering the entire teaching process. By implementing classroom questioning rules and post-class traceability management, they sought to rebuild collaborative learning based on student agency.

*“I randomly ask students to demonstrate good limb positioning for hemiplegic patients on the spot. This cuts off the time to rely on GenAI, forcing them to use their existing knowledge for expression and operation.” (P8 Neurology Nursing)*

Furthermore, to ensure transparency, students were required to submit a GenAI Usage Traceability Form with their assignments. This form mandated explicit labeling of which content referenced GenAI, the specific source, and personal modifications to GenAI conclusions, providing an objective basis for evaluating independent thinking. To address phantom collaboration in group work, some teachers utilized structured role division to reactivate group dynamics.

*“When working on pediatric nursing group assignments, I assign clear roles: a GenAI Searcher collects information, a Clinical Validator checks GenAI conclusions, a Plan Integrator, and a Reporter presents. Each member must submit a personal work record.” (P6 Pediatric Nursing)*

#### 3.3.3 Adapting AI-generated plans to clinical realities

Some DQNEs proactively designed deep interactive processes embedding GenAI into authentic or high-fidelity clinical scenarios.

*“I guide students to use GenAI to generate a wheelchair transfer plan for stroke patients. Then students practice on a simulator and record difficulties, like limb spasticity making GenAI steps impossible. I point out that GenAI did not consider spastic patient’ needs, while showing a video of real clinical operations*.” *(P1 Rehabilitation Nursing)*

They described deliberately situating AI-generated care plans within simulated clinical contexts to reveal discrepancies between standardized recommendations and practical nursing realities. They further reported that the use of de-identified clinical cases enabled students to compare AI-generated care plans with authentic nursing practices without raising ethical concerns.

“*If we use real cases, privacy is always a concern. So I prefer de-identified cases. Students can still see what actually happened in practice, but we don’t cross any ethical boundaries*” *(P11 Nursing Ethics)*

## 4 Discussion

This study employed a social constructivist framework to explore the pedagogical boundaries of GenAI in nursing education and the role reconstruction of DQNEs.

### 4.1 GenAI as a catalyst for cognitive conflict and offloading

Our results indicate that GenAI functions beyond a static information repository. It actively participates in the knowledge construction process of nursing undergraduate students by generating outputs that require verification and interpretation. This finding extends the concept of distributed cognition, wherein cognitive work is shared between human and technological agents (23). GenAI’s capacity positions it as what Wang et al. describe as a “partner in education” that dynamically responds to queries with variable outputs (24).

Generative AI facilitates cognitive offloading among nursing undergraduates by efficiently managing routine cognitive tasks, like retrieval of Wagner grading criteria. This pattern exemplifies what Smart (25) called “human-extended machine cognition” and aligns with research showing how technology supports learning by reducing cognitive load (25, 26). This release of working memory resources allows the pedagogical focus to shift to higher-order clinical reasoning. Unlike traditional nursing textbooks that present a single correct answer, GenAI often generates diverse or even conflicting outputs (27). When DQNEs leverage these discrepancies as pedagogical triggers, nursing undergraduates move beyond passive information reception to engage in collaborative dialogue and verification against clinical guidelines. In this capacity GenAI functions as a boundary that creates problem spaces for DQNEs guided collaborative inquiry, thereby supporting active meaning-making rather than mere information transmission.

### 4.2 Tensions between GenAI assistance and clinical learning

While GenAI promises enhanced efficiency in nursing education, our study reveals a tension between GenAI assistance and the social interactions necessary for developing nursing clinical expertise.

First, the use of GenAI raises concerns regarding academic integrity and professional ethics, particularly when GenAI output substitutes for required clinical engagement. Prior literature has warned that GenAI blurs boundaries between assistance and authorship, challenging traditional notions of authenticity in assessment (28). In nursing education, this issue extends beyond plagiarism to the erosion of professional responsibility. Clinical learning tasks are designed not only to generate data but to cultivate nursing professional identity. When such tasks are replaced by AI-generated narratives, nursing undergraduates bypass the moral and relational dimensions of practice that are essential to professional formation. This aligns with concerns in medical and nursing education that over-reliance on simulated or synthetic content may weaken learners’ sense of obligation toward real patients and clinical communities (29, 30).

Second, the integration of GenAI into collaborative learning may reshape group dynamics and produce unequal participation (31). This is problematic because collaborative learning requires all members to interact, for example by thinking aloud, giving explanations (32). Social constructivist theory emphasizes that knowledge emerges through interaction, negotiation, and the reconciliation of multiple perspectives. However, when GenAI outputs become the dominant reference point in group work, peer discussion may shift from substantive debate to surface-level endorsement. Rather than supporting collective meaning-making, GenAI risks becoming the default source of clinical knowledge, weakening students’ capacity to build clinical reasoning through social interaction.

Third, GenAI introduces the risk of epistemic displacement, whereby learners shift epistemic authority from clinical reasoning to GenAI responses (33). Scholarship has cautioned that GenAI produces confident outputs that mask uncertainty, potentially fostering over-trust among novice learners (34). In nursing education, this over-trust can lead students to treat GenAI recommendations as standards rather than suggestions requiring judgment. Clinical decision-making depends on patient-specific factors, comorbidities, protocols, and ethical considerations. When nursing undergraduates accept GenAI outputs without questioning their validity, they may develop simplified mental models unsuited to patient care. Such reasoning prioritizes textual coherence over adaptive judgment, conflicting with nursing education goals.

### 4.3 From transmitter to “contextual anchor”

The disruptions identified above require redefining the DQNEs’ professional role. In AI-integrated nursing education, the DQNEs’ value no longer lies in information delivery but in grounding algorithmic knowledge in clinical reality.

Double-qualified nursing educators must adopt a pedagogical stance that guides nursing undergraduates to interrogate GenAI outputs. The DQNEs’ role is to help nursing undergraduates understand not only what the GenAI suggests but why it suggests it and how it applies clinically. This approach aligns with constructivist pedagogy, which emphasizes that learning occurs through active questioning rather than passive acceptance of information (35). However, it also extends beyond traditional constructivism by requiring teachers to specifically address the epistemic challenges posed by algorithmic authority.

Double-qualified nursing educators also serve as contextual arbitrators who filter GenAI outputs through clinical reality. Faced with GenAI’s lack of nuance in cases like post-operative ambulation, teachers inject patient specific factors that algorithms cannot replicate. These include comorbidities, safety risks, and ethical considerations. This model represents a form of guided participation where teachers bridge the gap between algorithmic efficiency and clinical safety. Research in medical education has similarly argued that clinical supervisors must help trainees contextualize evidence-based guidelines within individual patient scenarios (36).

Sustaining this pedagogical model requires systemic institutional support to address the current state of governance gaps. Policies must move beyond punitive measures toward clear collaboration protocols. These protocols should mandate transparency in GenAI use to ensure that GenAI functions as a scaffold rather than a substitute for learning. For example, participants suggested implementing AI usage traceability forms that document how and when GenAI are consulted. This approach mirrors calls in medical education for transparent disclosure of GenAI assistance in clinical documentation (37, 38).

The risks associated with general GenAI highlight the need for nursing specific GenAI resources. Studies have documented that GenAI may provide outdated clinical guidelines or fail to account for discipline specific protocols (39). Institutions should invest in developing and validating nursing knowledge bases that integrate current evidence and clinical standards. This investment would ensure students train on accurate information rather than generic outputs.

Faculty development must evolve from basic digital literacy to pedagogical competence in GenAI contexts (40). Training should progress from foundational skills in validating GenAI outputs to advanced curriculum design that integrates GenAI with simulation and clinical reasoning exercises. This tiered approach recognizes that different levels of AI integration require different pedagogical capabilities. Literature on technology integration in health professions education has similarly advocated for faculty development that matches technological complexity with pedagogical expertise (41).

### 4.4 Strength and limitations

This study’s primary strength lies in its theory driven exploration of GenAI integration in nursing education through a social constructivist lens, with particular attention to the mediating role of DQNEs. Rather than evaluating GenAI as a standalone instructional tool, the study illuminates how teachers actively orchestrate human–AI collaboration to support knowledge co-construction and anchor GenAI outputs in clinical safety and professional judgment. By foregrounding DQNEs as contextual mediators, this study advances understanding of how pedagogical authority and clinical expertise remain central in AI-integrated learning environments and offers practical insights for faculty development in nursing education.

This study was conducted within a single educational context and relied primarily on teacher interviews, which may limit transferability. In addition, the analysis focused on pedagogical processes rather than measurable learning outcomes. Future studies could incorporate multi-site designs, student perspectives, and longitudinal approaches to further examine the educational impact of GenAI-integrated pedagogy.

## 5 Conclusion

Generative AI is reshaping the landscape of nursing education by acting as a powerful scaffold for inquiry. However, its integration carries the risk of hollowing out the social and practical interactions that define nursing competence. The future of nursing education lies in a Human-AI pedagogy, where DQNEs leverage GenAI for efficiency while rigorously maintaining their role as the guardians of clinical context.

## Data Availability

The raw data supporting the conclusions of this article will be made available by the authors, without undue reservation.
