# Supplementary material for: Role reconstruction among double-qualified nursing educators in the generative AI era: a qualitative study
Source: Front Med (Lausanne). 2026 Apr 10;13:1776845. doi: 10.3389/fmed.2026.1776845 (PMC13106064; doi:10.3389/fmed.2026.1776845)
Supplement: Supplementary file 1 [file Table_1.docx]

**Focus Goup Interview Guide**

# Supplementary Data

1. **How frequently do you observe students using GenAI in their nursing education, and in which learning contexts is GenAI use most common?**
2. **As a double-qualified nursing educator, what do you perceive as the core needs driving students’ self-initiated use of GenAI?**
3. **Have you provided any preliminary guidance or norms regarding students’ use of GenAI?**
4. **Have you ever used AI-generated cases as a “starting point” and guided students to revise or refine GenAI conclusions through literature review, simulated clinical practice, or group debate? If so, how did this approach contribute to the development of students’ clinical thinking?**
5. **How do you use GenAI tools to construct a triadic interactive learning environment involving clinical scenarios, students, and teachers?**
6. **When students use GenAI for personalized learning, do you supplement or adjust GenAI learning suggestions based on your own clinical experience? If so, how?**
7. **Do GenAI help you implement differentiated or stratified instruction for students with varying levels of learning ability?**
8. **When students become overly reliant on GenAI like focusing on AI-generated conclusions while neglecting clinical reasoning processes. How do you perceive and address this issue?**
9. **When students use GenAI in group learning, does GenAI sometimes dominate or replace interpersonal interaction? How do you interpret and respond to this phenomenon?**
10. **When students hold differing interpretations of GenAI outputs, how do you guide them toward sound clinical reasoning?**
11. **When assessing assignments or case analyses completed with GenAI assistance, do you encounter difficulties in distinguishing students’ independent thinking from GenAI content? How do you address this challenge?**
12. **If inappropriate GenAI use leads to learning deviations or misconceptions, how do you believe responsibility should be allocated? As a double-qualified nursing educator, what core guiding responsibilities do you see?**
13. **In your view, how should “GenAI ethical awareness” be cultivated in nursing education?**
14. **Given the widespread use of GenAI among students, which competencies do you believe double-qualified nursing educators need to further develop for future AI-integrated teaching?**
15. **What specific recommendations do you have for integrating student GenAI guidelines into undergraduate nursing curricula?**
